# Retraction: MicroRNA-137 Upregulation Increases Bladder Cancer Cell Proliferation and Invasion by Targeting PAQR3

**DOI:** 10.1371/journal.pone.0269903

**Published:** 2022-06-08

**Authors:** The PLOS ONE Editors

Following the publication of this article [1], similarities were noted between this article and articles submitted by other research groups, including [2–10], of which one article [10] was previously retracted [11].

Similarities included the following figures, which appear to fully or partially overlap, despite being published in different articles and representing different conditions:

- inhibitor panel in Fig 3E of [1] and miR-217-mimic panel in Fig 2D of [5].
- pCDNA-PAQR3 scamble panel in Fig 5E of [1] and the BRD7 panel in Fig 5D of [7] in grayscale.
- PAQR3 panel in Fig 4D of [1], L1CAM panel in Fig 4D of [4], WASF3 panel in Fig 3D of [5], and BRD7 panel in Fig 4C of [7].
- Lanes 1–2 of the GAPDH panel in Fig 4D of [1], and lanes 2–3 of the GAPDH panel in Fig 3D of [6].

Although the corresponding author initially replied to acknowledge receipt of our message, *PLOS ONE* did not receive responses to the queries regarding these concerns by the end of the original deadline and extension.

The unresolved concerns call into question the validity and provenance of the reported results, and the adherence of this article to the PLOS Authorship policy. Therefore, the *PLOS ONE* Editors retract this article [1].

All authors did not comment on the retraction decision, did not respond directly or could not be reached.
